# Effects of cyclic compression on the mechanical properties and calcification process of immature chick bone tissue in culture

**DOI:** 10.1016/j.bonr.2017.04.002

**Published:** 2017-04-04

**Authors:** Eijiro Maeda, Masashi Nakagaki, Katsuhisa Ichikawa, Kazuaki Nagayama, Takeo Matsumoto

**Affiliations:** a Biomechanics Laboratory, Department of Mechanical Science and Engineering, Graduate School of Engineering, Nagoya University, Nagoya, Japan; b Biomechanics Laboratory, Department of Mechanical Engineering, Nagoya Institute of Technology, Nagoya, Japan; c Micro-Nano Biomechanics Laboratory, Department of Intelligent Systems Engineering, Ibaraki University, Hitachi, Japan

**Keywords:** Cyclic compression, Mechanical loading, Elastic modulus, Calcification, Ex vivo culture

## Abstract

Contribution of mechanical loading to tissue growth during both the development and post-natal maturation is of a particular interest, as its understanding would be important to strategies in bone tissue engineering and regenerative medicine. The present study has been performed to investigate how immature bone responds to mechanical loading using an ex vivo culture system. A slice of the tibia, with the thickness of 3 mm, was obtained from 0-day-old chick. For the ex vivo culture experiment in conjunction with cyclic compressive loading, we developed a custom-made, bioreactor system where both the load and the deformation applied to the specimen was recorded. Cyclic compression, with an amplitude of 0.3 N corresponding to 1 to 2% compressive strain, was applied to immature bone specimen during a 3-day culture period at an overall loading rate 3–4 cycles/min, in the presence of β-glycerol phosphate and dexamethasone in culture medium. The stress-strain relationship was obtained at the beginning and the end of the culture experiment. In addition, analyses for alkaline phosphate release, cell viability and tissue calcification were also performed. It was exhibited that elastic moduli of bone slices were significantly elevated at the end of the 3-day culture in the presence of cyclic compression, which was a similar phenomenon to significant elevation of the elastic moduli of bone tissue by the maturation from 0-day old to 3-day old. By contrast, no significant changes in the moduli were observed in the absence of cyclic compression or in deactivated, cell-free samples. The increases in the moduli were coincided with the increase in calcified area in the bone samples. It was confirmed that immature bone can respond to compressive loading in vitro and demonstrate the growth of bone matrix, similar to natural, in vivo maturation. The elevation of the elastic moduli was attributable to the increased calcified area and the realignment of collagen fibers parallel to the loading direction. The ex vivo loading system established here can be further applied to study responses to mechanical loading in osteogenesis as well as callus maturation for better understanding of factors to consider in successful bone regeneration with mechanical factors.

## Highlights

• We examined how immature bone responds to cyclic compressive loading, using a newly established tissue loading system.
• Loading culture of 0-day-old chick tibia slice for 3 days significantly increased elastic moduli and calcified area.
• The moduli and calcified area after 3-day loading culture were comparable with those from 3-day-old chick tibia.
• No increases in the moduli or in calcified area were observed in non-loaded samples and deactivated samples.
• It was suggested that cyclic compressive loading is an essential factor of normal bone growth in ex vivo bone culture.

## 1 Introduction

It is well established that both tensile and compressive loadings are applied to bone by body movement, which in turn generates hydrostatic pressure and shear stress at a cellular level (Curry, 2002). Bone responds to changes in its mechanical environments; the frequency and the amplitude of such loadings can be changed due to maturation, physical activity, and injury. In the functional adaptation, the balance between bone formation and bone resorption is regulated. The molecular mechanism by which bone adapts to loading became clear in recent years (Robling et al., 2006).

Contribution of mechanical loading on tissue growth during both the development and post-natal maturation is of a particular interest, as its understanding would be important to strategies in bone tissue engineering and regenerative medicine. There have been several studies on responses of bone tissue to mechanical loading in immature animals (Matsuda et al., 1986, Judex and Zernicke, 2000a, Judex and Zernicke, 2000b). It was demonstrated that running immature rooster for 15 min/day induced no enhancing effects on the growth of cortical bone in legs (Judex & Zernicke, 2000a) and for 1 h/day provided negative effects on mechanical properties and morphology (Matsuda et al., 1986), whereas high impact loading with high strain rate provided stimulatory effects on bone growth (Judex & Zernicke, 2000b). In contrast, disuse of legs in immature canine inhibited bone growth, but did not induce bone resorption (Uhthoff & Jaworski, 1978). However, because bone growth/formation and resorption are regulated not only by mechanical loading but also by other factors such as hormones and cytokines, it is difficult to isolate effects of mechanical loading in such in vivo models. Indeed, during maturation, bone tissue undergoes drastic changes in the shape and the structure, including bone matrix calcification, collagen cross-linking, and formation of Haversian systems, and subsequently in mechanical properties (Curry, 2002, Isaksson et al., 2010a, Isaksson et al., 2010b). To overcome difficulties in studying the effects of mechanical loading alone in bone adaptation, experimental models of ex vivo tissue culture in conjunction with an application of controlled mechanical loading have been utilized (for example, (Jones et al., 2003) for studying adult bone tissue remodeling). For understanding immature bone growth, cyclic hydrostatic pressure was applied to isolated whole fetal femur from chick in a culture condition, and bone formation, mineralization and associated change in gene expressions were examined (Henstock et al., 2013). However, there has been no study to investigate effects of cyclic compressive loading on the growth of immature bones using such ex vivo model.

Therefore, the present study has been performed to 1) develop a new, custom-made tissue loading system for ex vivo bone explant culture, enabling to apply cyclic compressive loading to the explants, and 2) investigate how immature bone responds to mechanical loading.

## 2 Materials and methods

### 2.1 Specimens

Immature bone tissues obtained from the tibias of 0-day-old chick (white leghorn) were used in the present study. The animals were sacrificed with CO_2_ gas. Following sterilization of both legs using 70% ethanol, the tibias were obtained in a sterile condition in a clean bench. A bone slice with a thickness of 2–3 mm was obtained with a scalpel in a plane perpendicular to the tibial axis at the position 3 mm from the proximal end of each tibia. The slices were then cultured in experimental conditions described below for 3 days in an osteogenic medium, which consisted of BGJb (Gibco, USA) supplemented with 10% fetal calf serum (ICN, USA), 75 μg/ml of ascorbic acid (Wako, Japan), 5 mM β-glycerol phosphate (Wako) and 0.1 μM dexamethasone (Wako).

For examining effects of cyclic compression, we set four experimental groups: cyclic compression culture (CCC), static culture (SC) without compressive loading, cyclic compression culture with deactivated bone slice (dCCC) and static culture with deactivated bone slice (dSC). The deactivated bone specimens were prepared by repeating freezing freshly obtained bone slices in liquid nitrogen and thawing at room temperature for a few times to kill all the bone cells in the samples. In addition, bone slices tested for mechanical properties and histology immediately after sacrifice at 0-day-old and 3-day-old were assigned as controls (C0 and C3, respectively).

### 2.2 Cyclic compression culture

**Fig. 1:**
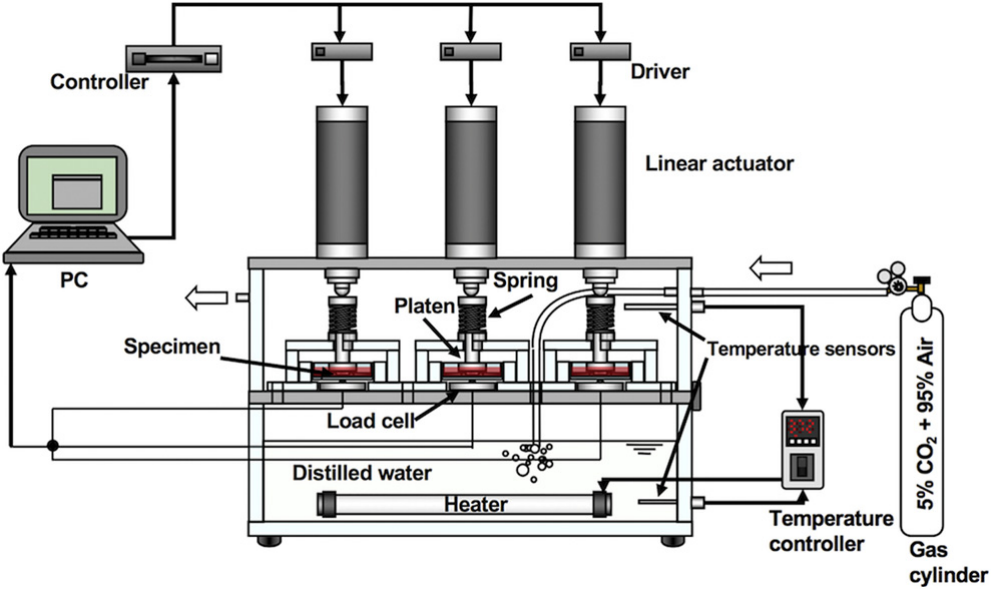
Schematic illustration of the ex vivo bone loading system that we have newly developed. Three specimens can be cultured with cyclic compressive loading, and another three specimens can be cultured in non-loading, static culture condition in a single experimental run.

**Fig. 2:**
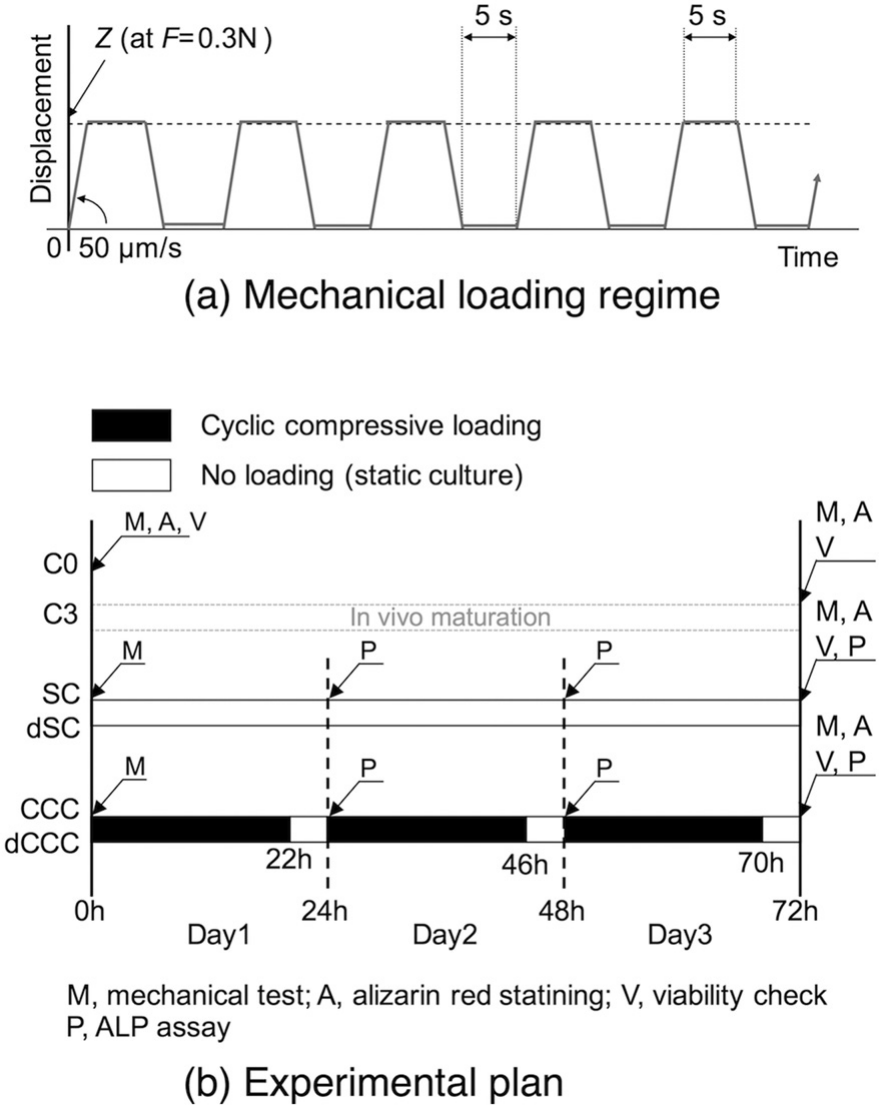
(a) Schematic of the mechanical loading regime adopted in the present study. (b) Experimental plan in the present study. C0, day 0 control; C3, day 3 control; SC, static culture; CCC, cyclic compression culture; dSC, deactivated static culture; dCCC, deactivated cyclic compression culture.

We have developed the apparatus for the cyclic compression culture, which is schematically shown in Fig. 1. Three bone slices obtained from the right tibias were cultured simultaneously under cyclic compression along with their contralateral static controls under a standard culture condition (37 °C, 100% humidity, 5% CO_2_ and 95% air) in an acrylic box. Each of the specimens in a 35-mm culture dish was compressed cyclically in the direction of tibial axis for 3 days with a linear actuator (MAS-23, Chiba Precision, Japan) via a platen. The magnitude of compressive load applied to each of loaded specimens was monitored with a load cell (TC-USR-23, TEAC, Japan) placed under the dish of the loaded bone slice. To apply mechanical stimulation to the slices, the following loading regime in a trapezoidal waveform was adopted: loading at the rate of 50 μm/s until the load reached 0.3 N, holding at 0.3 N for 5 s, unloading to 0.001 N at the same rate and 5 s resting period at 0.001 N, resulting in an overall loading rate 3–4 cycles/min depending on the stiffness of the slice (Fig. 2(a)). The cyclic compression was provided for 22 h, followed by a 2 h resting period for changing the medium and correcting the sample position (Fig. 2(b)). This 24 h loading protocol was repeated three times during the culture period, resulting in a total of a 3-day culture period. A custom-built operating program (LabVIEW, National Instruments, USA) was used to control the actuators and collect the data from load cells. For samples assigned to SC, bone slices were placed in 35-mm culture dishes and set in the apparatus in the same fashion with the samples for CCC, but without the platens. The slices were cultured without external mechanical stimulation for three days along with the samples of CCC. In addition, in separate sets of the experiment, deactivated bone slices were also cultured with or without cyclic compressive loading for 3 days as described above.

### 2.3 Mechanical test

Mechanical properties of the slices assigned to the 3-day culture experiment were determined with a compression test performed before and after the 3-day culture period. The specimens were compressed at the rate of 0.5 μm/s up to 0.5 N to obtain nominal stress-strain curves. Nominal stress *σ* and strain *ε* was calculated as *σ = F/A*_0_ and *ε = x/t* where *F* and *x* are the load and displacement, respectively, applied to the slice, and *A*_0_ and *t* the cross-sectional area and the thickness of the slice after the culture, respectively. *A*_0_ of each slice was determined through an image analysis of a photomicrograph of the slice obtained with a stereoscopic microscope (SZH10-111, Olympus, Japan), and *t* was measured with a caliper. To reduce ambiguity of the origin of the curve, zero strain was taken as *F* = 0.003 N, which corresponded to approximately compressive stress at 0.004 MPa. Apparent elastic modulus was determined from the slope of each of the following four stress ranges: between 0.004 and 0.01 MPa (*E*_0_), between 0.01 and 0.02 MPa (*E*_1_), between 0.02 and 0.03 MPa (*E*_2_) and between 0.03 and 0.04 MPa (*E*_3_).

### 2.4 Alkaline phosphatase activity assay

In selected samples assigned to CCC and SC, culture media were collected at the end of 22 h loading period on each day. Alkaline phosphatase (ALP) activity was determined using a commercially available kit (WK27404401, Wako), by measuring the hydrolysis of *p*-nitrophenyl phosphatase. The product was quantified by spectrophotometry at 405 nm (U-1800, Hitachi, Japan). ALP activity measured in freshly prepared culture medium was used as control.

### 2.5 Viability test

**Fig. 3:**
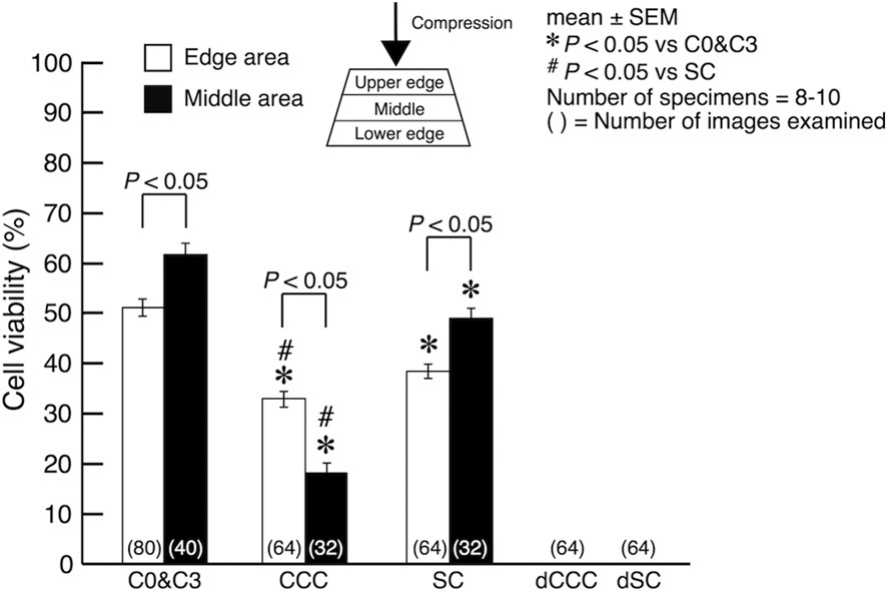
Cell viability of each experimental group. C0&C3 is from pooled data separately determined from C0 and C3 specimens. Statistical significances are indicated as *P < 0.05 vs C0&C3 and ^#^*P* < 0.05 vs SC.

At the end of the 3-day culture period, selected eight specimens were embedded in paraffin and sliced in the direction parallel to the tibial axis with a microslicer (DTK-1000, Dosaka-EM, Japan) to obtain 200-μm-thick slices at the center of the specimen. The sections were stained with 2 μM of calcein-AM and 4 μM of ethidium homodimer-III in PBS (both from Biotium, USA) for 45 min at room temperature in the dark. The former was used to stain viable cells, while the latter to stain dead cells. The stained section was observed with a fluorescence microscope (IX-71, Olympus, Japan). For the observation and cell counting, we defined three areas in the section as schematically drawn in Fig. 3; namely upper edge area, middle area and lower edge area. In each area, four of a 300 μm × 300 μm region of interest was randomly selected to count the number of viable and dead cells. The cell viability was calculated in each region by dividing the number of viable cells by the total number of cells. Data of the upper edge area and the lower edge area from the eight specimens (a total of 64 viability values) were pooled and an average value of these data was calculated as a representative viability of these areas. The viability for the middle area was determined as an average from the pooled data from the eight specimens (a total of 32 viability values). Data obtained from C0 and C3 samples (five specimens for each group) were pooled to represent the viability of freshly isolated bone slices (denoted as C0&C3). The viability of the edge areas and the middle area was calculated in the same fashion as described above.

### 2.6 Measurement of calcification area

Specimens assigned to the measurement of calcification were also embedded in paraffin at the end of the culture period, and sectioned to 200 μm in thickness in the direction perpendicular to the tibial axis with the microslicer. The slices were stained with 1% Alizarin Red S (Wako) and observed on an inverted microscope (IX-71, Olympus, Japan) to measure calcified area. The fraction of calcification area to the total area of the bone specimen was calculated using ImageJ (NIH, USA).

### 2.7 Statistical analysis

For the differences in the cross-sectional area and the thickness, Dunnett test was used to compare C0 with C3, CCC, SC, dCCC and dSC. Student's *t*-test was also used to compare CCC with SC, and dCCC with dSC.

Regarding the differences in strain level at every 0.005 MPa from 0.01 MPa to 0.04 MPa in the stress-strain relationships, statistically significant differences were analyzed with Student's *t*-tests at each stress level between C0 and C3, and before and after the 3-day culture in CCC, SC, dCCC and dSC.

For elastic moduli, Dunnett test was performed to compare C0 with CCC (before), SC (before), dCCC (before) and dSC (before), and to compare C3 with CCC (after), SC (after), dCCC (after) and dSC (after). Differences between before and after the 3-day period, including between C0 and C3, those between cyclic compression culture and static culture, and those between viable and dead samples were assessed with Student's *t*-tests.

For ALP activity, comparisons of the level of CCC and SC on each day to that of fresh medium was performed with Dunnett test. Comparisons among days 1, 2 and 3 (for CCC and SC) were carried out using one-way ANOVA, followed by Tukey multiple comparison tests. Further, CCC and SC were compared with Student's *t*-test on each day.

For cell viability, the difference between C0&C3 and CCC as well as SC was assessed using Dunnett test in both edge and middle areas. In addition, the differences between edge area and middle area as well as between CCC and SC in each area were analyzed using Student's *t*-tests.

For the fraction of calcified area, comparison of C3, CCC and SC to C0 was carried out using Dunnett test. Differences among C3, CCC and SC were examined with one-way ANOVA, followed by Tukey multiple comparison tests. Pearson's correlation coefficient was determined through a linear regression analysis between the calcified area fraction and elastic modulus.

All the analyses were performed using a statistical computing language R (version 3.3.2), and significance level was at *P* < 0.05.

## 3 Results

### 3.1 Dimensions of bone samples

The cross-sectional area of bone samples obtained and measured at 0-day-old (C0) was 6.8 ± 1.1 mm^2^ (mean ± SEM, *n* = 15), which was significantly increased to 8.9 ± 1.5 mm^2^ (*n* = 15) at 3-day-old (C3) (Table 1). The area was also increased from the C0 level following the 3-day culture period in CCC, SC, dCCC and dSC; however, a significant difference was only observed between dCCC and C0. No significant differences were also observed between CCC and SC, and dCCC and dSC. The thickness of the C0 sample was 2.46 ± 0.03 mm (*n* = 15), and this was not changed much following the 3-day maturation as well as at the end of the 3-day culture period.

**Table 1:** Summary of dimensions of bone disc specimens after culture.

	C0 (15)	C3 (15)	CCC (15)	SC (15)	dCCC (10)	dSC (10)
Cross-sectional area (mm^2^)	6.8 ± 0.3	8.9 ± 0.3#	8.1 ± 0.5	7.7 ± 0.3	9.0 ± 0.6#	7.9 ± 0.8
Thickness (mm)	2.46 ± 0.03	2.57 ± 0.07	2.39 ± 0.03	2.38 ± 0.04	2.46 ± 0.05	2.65 ± 0.08

( ) = number of specimens; mean ± SEM.

# *P* < 0.05 vs. C0.

### 3.2 Specimen viability

Fig. 3 shows the summary of cell viability assessments at the edge and the middle areas in each group. The cell viability in the control (C0&C3) was 50.9 ± 1.7% (*n* = 80) and 61.5 ± 2.3% (*n* = 40) in the edge and the middle areas, respectively. The viability of cultured samples was decreased significantly in both areas. Indeed, the viability in CCC was 32.6 ± 1.5% (*n* = 64) and 17.7 ± 2.3% (*n* = 32) in the edge and the middle areas, respectively, and that in SC was 38.0 ± 1.4% (*n* = 64) and 48.7 ± 2.0% (*n* = 32), respectively. In addition, the viability of SC was significantly higher than that of CCC in both areas. It was also demonstrated that the viability in the middle area was significantly higher than that in the edge areas in the control and SC, while the trend was opposite in CCC. For dCCC and dSC, it was confirmed that no cells were viable in these deactivated samples.

### 3.3 Mechanical properties of bone samples

**Fig. 4:**
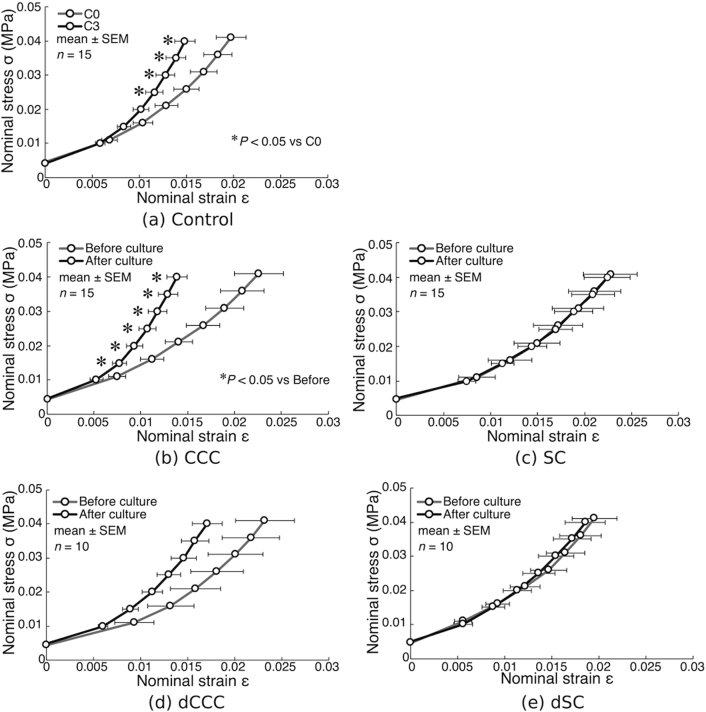
Changes in stress-strain relationships during maturation (a) and before and after the 3-day culture (b–e). Statistical significances are indicated as *P < 0.05 vs C0 (a) or Before (b).

Nominal stress-strain curve demonstrated a nonlinear behavior, increasing the slope of the curve with increase in compressive strain. In the control samples, the curve became steepened from C0 to C3 (Fig. 4(a)). The similar trend of curve steepening following the 3-day culture period was observed in CCC (Fig. 4(b)). Indeed, the strain level was significantly less in C3 than in C0 in the range of stress between 0.025 and 0.04 MPa, and in CCC after the culture than that before the culture in the range between 0.015 and 0.04 MPa. By contrast, the relationship exhibited no changes between before and after of the 3-day culture in SC (Fig. 4(c)). In deactivated samples, the stress-strain curve did not change significantly following the 3-day cyclic compression culture either in dCCC (Fig. 4(d)) or dSC (Fig. 4(e)), although the curve looked steepened in dCCC.

**Fig. 5:**
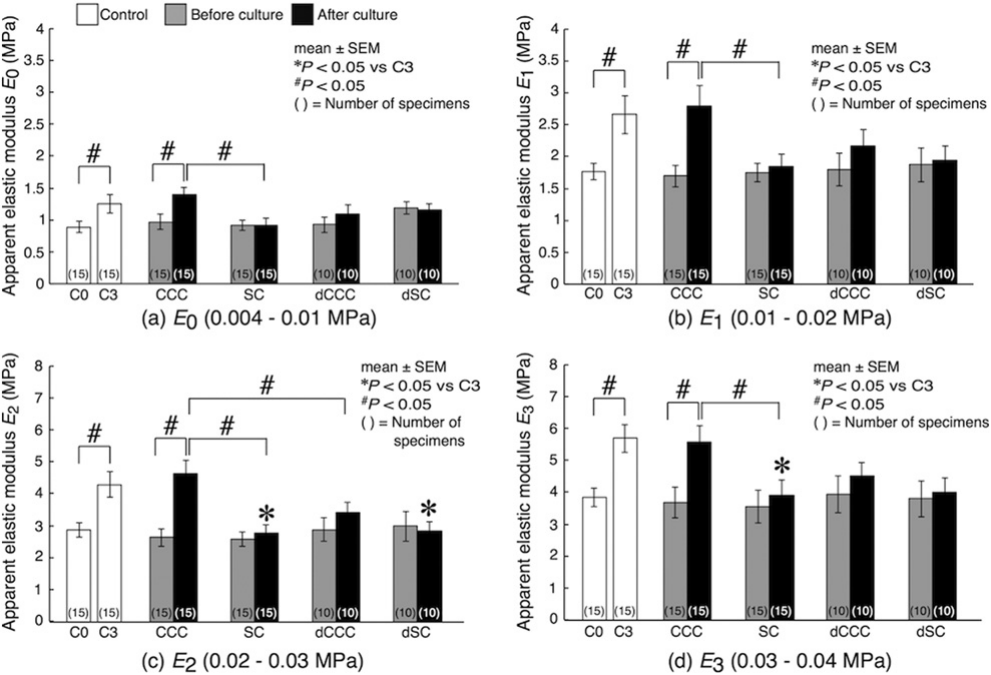
Changes in apparent elastic moduli following culture. The apparent moduli were determined in 4 stress ranges from the stress-strain relationships shown in Fig. 4. Statistical significances are indicated as *P < 0.05 vs C3 and ^#^*P* < 0.05.

Further investigations of stress-strain relationships by determining apparent elastic moduli revealed strengthening effects of cyclic compressive loading on cultured bone explants. For control samples, all the moduli (*E*_0_, *E*_1_, *E*_2_ and *E*_3_) increased significantly from C0 to C3 during the 3-day maturation (Fig. 5). Similarly, the 3-day cyclic compression culture (CCC group, after the culture) resulted in in significant increases in all the moduli, reaching to the similar levels with C3. By contrast, the 3-day static culture (SC) induced no changes in the moduli between before and after the culture. Significant differences were observed in all the moduli between CCC and SC after the culture. There was also a significant difference in *E*_2_ and *E*_3_ between C3 and SC after the culture. In deactivated samples, dCCC exhibited increases in the moduli by the cyclic compression culture, but the changes were not statistically significant. There were no changes observed in dSC in the moduli between before and after the culture. *E*_2_ in dSC after the culture was significantly lower than that in C3 samples.

### 3.4 Alkaline phosphatase activity

**Fig. 6:**
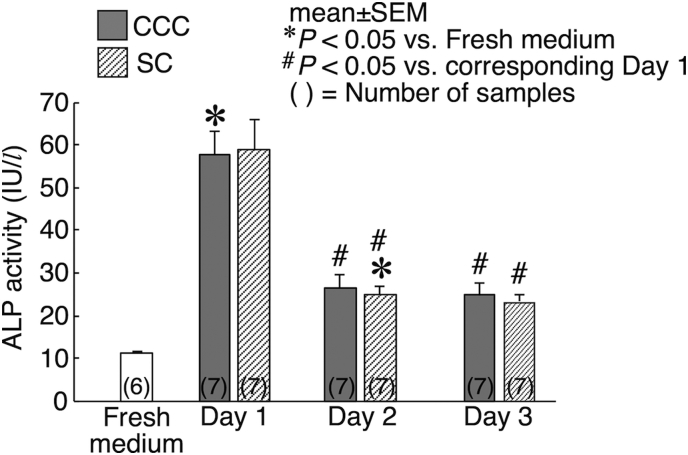
Alkaline phosphatase activity within the culture media collected at the end of the culture on day 1, day 2 and day 3. Data obtained from fresh culture medium was used as control. Statistical significances are indicated as *P < 0.05 vs Fresh medium and ^#^*P* < 0.05 vs corresponding Day 1 level.

ALP activity was markedly high in both CCC and SC on day 1, followed by a decreased level of activity on days 2 and 3 (Fig. 6). Statistically significant differences to the level in fresh medium were observed in CCC on day 1 and SC on day 2. The decreases from the level on day 1 to the levels on days 2 and 3 were statistically significant in both CCC and SC. Nonetheless, there were no significant differences between CCC and SC.

### 3.5 Evaluation of bone calcification

**Fig. 7:**
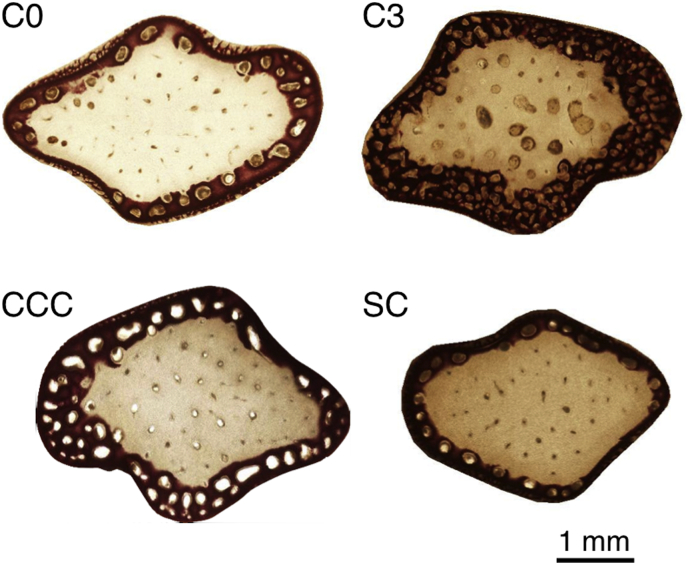
Representative photomicrographs of the cross section of bone specimens, stained with Alizarin Red S. Regions stained in red corresponded to calcified bone tissue, and non-stained, white regions corresponded to osteoid or cartilage tissue.

In C0, there was a thin layer of calcified area in the outer edge of the sample cross section (Fig. 7). A number of cavities were also observed. Non-stained area in the middle corresponded to osteoid or cartilage tissue. In C3, the bone itself enlarged and the calcified layer was also thickened. The thickening of calcified are was also observed in CCC, which appeared similar to C3 sample. However, the calcification in SC remained similar to C0.

**Fig. 8:**
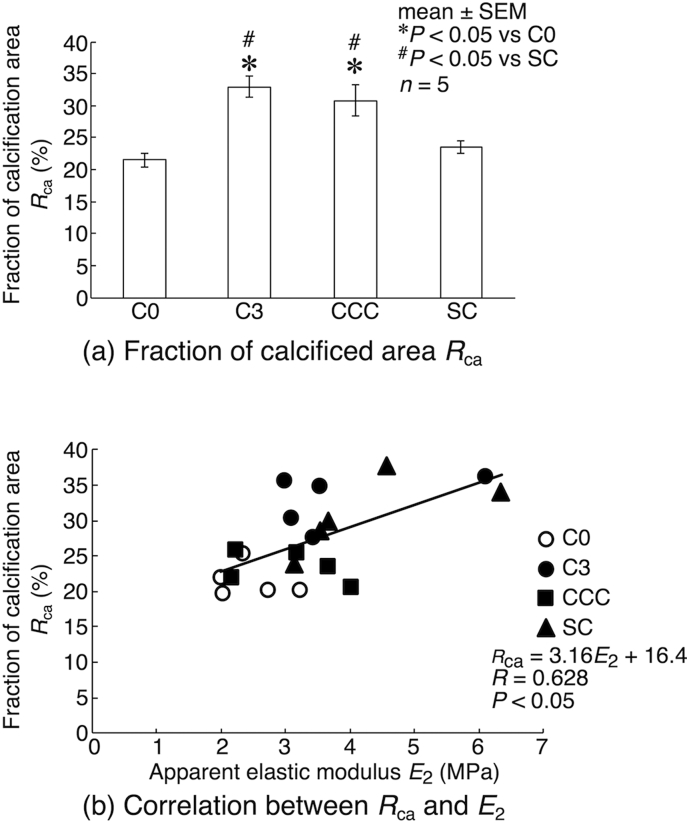
(a) The fraction of calcified area to the total cross-sectional area of bone samples. Statistical significances are indicated as *P < 0.05 vs C0 and ^#^*P* < 0.05 vs SC. (b) Linear regression analysis revealed that there was a significant correlation between the fraction of calcification area and apparent elastic modulus.

Quantitative analysis of these images show that the fraction of calcified area to the total cross-sectional area of bone specimens was 21.4 ± 1.0% (*n* = 5) in C0 (Fig. 8(a)). The calcification progressed during the 3-day in vivo maturation, resulting in significantly larger area fraction in C3 (32.9 ± 1.6%, *n* = 5). With the application of cyclic compressive loading, the area fraction was 30.8 ± 2.3% (*n* = 5) in CCC, which was also significantly larger than C0 level and was comparable to the level of C3. On the other hand, the area fraction in SC (23.4 ± 0.9%, *n* = 5) was at a similar level with C0, and was also significantly lower than that of C3 and CCC. A positive correlation between elastic modulus (pooled from C0, C3, CCC and SC data) and the fraction of calcified area in corresponding samples was observed in *E*_0_, *E*_1_, *E*_2_ and *E*_3_. Indeed, statistically significant correlations were obtained in *E*_1_, *E*_2_ and *E*_3_ (data were only shown for *E*_2_) (Fig. 8(b)).

## 4 Discussion

The present study was performed to examine effects of cyclic compressive loading on mechanical properties of cultured immature bone slices from the tibia of 0-day-old chicks. It was demonstrated that the application of cyclic compressive loading for three days elevated elastic moduli of the bone slices, to the level comparable to that obtained from the tibia of 3-day-old chicks. In addition, the fraction of calcified area was also similar between CCC at the end of the culture period and C3. On the other hand, static culture for three days without the application of cyclic compressive loading resulted in no alterations in the sample size, elastic moduli and the fraction of calcified area from the levels of 0-day-old chicks. Accordingly, these findings suggest that the application of cyclic compressive loading at the current level provided positive, anabolic effects on both mechanical properties and histology of immature bone tissues.

It was confirmed from the data recorded from load cells that cyclic compressive loading was successfully applied to cultured bone specimens. The magnitude of the loading was 0.3 N, which was slightly lower than the force generated by the body weight of the newborn chicks (40 to 50 gf), and thus could be assumed at a physiological level. This corresponded to the application of approximately 1 to 2% compressive strain (10,000 to 20,000 με) (Fig. 4). However, according to “mechanostat” theory by Frost (Frost, 2003, Duncan and Turner, 1995, Rosa et al., 2015), the range of strain inducing anabolic and adaptive bone modeling is between 1500 and 3000 με (0.15 and 0.3% strain). Thus, the current loading amplitude could have been more than stimulatory and rather pathological. It has been exhibited that a moderately intensive training (running on a treadmill with 70–80% of maximum O_2_ consumption) of immature 3-week-old chicks reduced stiffness and yield stress of leg cortical bone (Matsuda et al., 1986), indicating that overloading induced a pathological remodeling. The current findings showed the increase in the elastic moduli of newborn chick bones even in the presence of loading with such high strain level, suggesting that the cell in newborn chick bones may have a wider capacity of strain level to sense mechanical loading as anabolic stimulation.

Cell viability within the explants was already 50 to 60% in C0 and C3 samples. This may indicate that bone samples were given some damages at the time of the sample preparation, and a part of cell population underwent damaged cell death (necrosis). The 3-day culture period further reduced cell viability to 17 to 33% in CCC and 38 to 48% in SC. The lowering ALP activity in CCC and SC samples during the 3-day culture may reflect the decreasing in the cell viability. The loss of viable cells during bone explant culture has also been reported (Takai et al., 2004, Mann et al., 2006). In the case of the application of cyclic compressive strain, the cell viability within human trabecular bone explants following a 3-day culture with cyclic strain of 0.003% at 1 Hz resulted in 69.5%, which was reduced to 37.5% without the stimulation (Mann et al., 2006). Such loss of the viability has been attributed to hypoxic environment in the center of the explant, due to the lack of diffusion of medium (Mann et al., 2006). Indeed, a perfusion culture of trabecular bone explants from rat femur maintained 70% cellularity after 14 days of ex vivo culture (Davidson et al., 2012). Although cyclic compressive loading was expected to enhance diffusion of culture medium to supply nutrients and oxygen to the center of the bone explant, it was exhibited in the present study that the viability in the middle area of the explant was lower than that in the edge areas in CCC. This may also be attributable to the lack of perfusion of culture medium. Because the upper and the lower surface of the loaded explant was kept contacting to the platen and the bottom surface of the culture dish, respectively, this could have blocked diffusion of the medium from these surfaces, resulting in hypoxia and malnutrition, and subsequent cell death. It is interesting that ALP activity in CCC maintained the level similar to that in SC, even with the lower cell viability. This may be attributable to that osteoblasts in SC downregulated ALP synthesis due to the absence of mechanical stimulation, or that the cells in CCC, even with the fewer number than in SC, upregulated ALP synthesis by cyclic compression to compensate the cell loss.

Mechanical and compositional analysis of the growth of immature (or newborn) cortical bones during maturation in vivo has been reported in human (Hirsch & Evans, 1965), baboon (Keller et al., 1985) and rabbit (Isaksson et al., 2010a, Isaksson et al., 2010b), which involved increases in tissue elasticity (Isaksson et al., 2010a, Isaksson et al., 2010b, Hirsch and Evans, 1965, Keller et al., 1985) as well as collagen content, collagen cross-links and mineralization (Isaksson et al., 2010a, Turunen et al., 2011, Turunen et al., 2010), and decreases in fatigue resistance (Keller et al., 1985) and viscoelasticity (Isaksson et al., 2010b). In in vitro studies in conjunction with cyclic mechanical loading, an increase in elastic modulus of matured bovine trabecular bone cores (Vivanco et al., 2013) and new bone formation in fetal chick long bones (Henstock et al., 2013) have also been demonstrated. The present findings are consistent with these previous findings, indicating that the current experimental settings as well as mechanical stimulation regime provided the mechanical environment similar to spontaneous, in vivo maturation, which may reflect increasing body weight and/or physical activity. In addition, the increase in the elastic moduli by mechanical loading culture observed in the present study can be attributed to responses of osteocytes and osteoblasts within the explants to cyclic compression, such as upregulations of collagen synthesis and tissue mineralization. In static culture (SC), there were no changes in the elastic moduli, the fraction of calcified area and ALP activity, although the cell viability was better when compared to CCC. This may indicate that the development of immature cortical bone was impaired in the absence of mechanical loading, as has been suggested (Uhthoff and Jaworski, 1978, Marino et al., 2016).

In contrast, the explants in dCCC also slightly increased the elastic moduli, and deformed less to compressive stress when compared to its corresponding samples before the culture, although none of these changes were significant. This may be due to that cyclic compression reorganize collagen network of the cortical bone. Newborn cortical bone consists of woven-fibered bone with collagen fibers running in random directions (Carter & Spengler, 1978), and thus possess low elasticity and strength (Isaksson et al., 2010a, Isaksson et al., 2010b). It is, therefore, presumed that cyclic compression aligned the collagen fibers parallel to the loading direction, and provided the tissue with elasticity and strength sufficient to adapt to the surrounding mechanical environment.

Calcification of cultured bone was only evident in the presence of cyclic compression, which suggests that cyclic compressive loading enhanced bone calcification. Matrix calcification is thought to proceed in two phases (Anderson, 1995, Anderson, 2003); phase 1 is initiated by cells (osteoblasts in bone) releasing matrix vesicles containing substrates and enzymes to generate the first crystals of calcium phosphate, and phase 2 involves growing to larger crystals outside the vesicles and mineralizing collagen matrix. The cells also regulate extracellular ionic conditions and matrix composition in the second phase, which can proceed independently of ALP (Anderson, 1995). In the present study, because approximately 20% of the cross-sectional area of cortical bone specimen was calcified in newborn chick (Fig. 7, Fig. 8), there could have been both phases 1 and 2 mineralization during the 3-day culture period. In a previous study culturing a whole fetal chick femur, cortical thickening and calcification were observed when cultured in osteogenic medium, including β-glycerol phosphate and dexamethasone, even in the absence of mechanical stimulation (Henstock et al., 2013). This was further enhanced in the presence of cyclic hydrostatic pressure. However, only a slight increase was observed when cultured in non-osteogenic medium, even in the presence of cyclic hydrostatic pressure. As the osteogenic factors (β-glycerol phosphate and dexamethasone) are effective in phase 2 calcification, phase 2 calcification seems to be predominant in the post-natal bone maturation. Thus, the increased calcification in the present study demonstrated a synergetic effect of the osteogenic factors in the medium and cyclic compressive loading, where cyclic compression possibly stimulated osteoblasts to regulate extracellular ionic condition to be in favor of calcification and/or to upregulate collagen synthesis to provide more substrates for calcification. In the case of SC, the calcified area was not greatly increased, possibly because there were no such upregulated functions of osteoblasts. These findings give us a new hypothesis that cyclic compressive loading stimulates collagen synthesis from osteoblasts, aligns existing and new collagen fibers to the loading direction, creating more organized interfibrillar spaces for calcification, and enhances tissue calcification along with the direction of collagen fibers to provide mechanical integrity to bone tissue. This hypothesis will be tested in our future work. Nonetheless, it should be stated that the current experimental techniques and the data obtained cannot distinguish the two possible mechanisms of promoting tissue calcification by mechanical loading; one is the passive delivery of proteins and minerals facilitating mineralization from the medium to bone matrix by mechanical loading, and the other is biological effects of mechanical loading such as cellular regulation of extracellular environment and upregulation of collagen synthesis.

In addition, the thickening as well as the calcification of bone slice was appeared to progress on the endocortical surface (inward), rather than periosteally (outward). However, the results obtained in the present study provide no evidences of the direction of the tissue growth. Thus, this also highlights the need to study the direction of tissue growth and calcification in detail.

One of advantages of the present experimental model using newborn bone explants is that the variability between samples in immature animals are less that in matured animals (Hirsch & Evans, 1965), partly because that there is no need to consider the history of injuries, differences in diet and body sizes. This is also beneficial to prepare samples with almost the same size. However, immature bone slice is not the sole application of the loading system established. With a high precision control of load and deformation applied to the specimens and the capabilities of measuring mechanical properties of the specimens during culture, the current system is also suitable to study responses of embryonic bone tissue as well as tissues regenerated in a bone fracture (callus). The understanding of the development of bone is essential to the success in bone regenerative medicine. By studying the similarities and the differences in responses to mechanical loading between osteogenesis and callus maturation from biomechanical point of view, we could understand factors that we must consider in mechanically induced tissue regeneration in bone.

In conclusion, the present study developed a custom-made cyclic compression system. Using the system, the application of cyclic compressive loading to the explant from the tibia of newborn chick successfully induced the increase in the elastic moduli and calcified area. These findings suggest that the bone maturation by cyclic compression in vitro was the similar phenomena with natural, in vivo maturation. Therefore, the maturation of immature cortical bone requires mechanical stimulation.

## Conflict of interests

The authors have no conflicts of interests.
